# ZnO Quantum Dots Modified by pH-Activated Charge-Reversal Polymer for Tumor Targeted Drug Delivery

**DOI:** 10.3390/polym10111272

**Published:** 2018-11-15

**Authors:** Yifan Wang, Liang He, Bing Yu, Yang Chen, Youqing Shen, Hailin Cong

**Affiliations:** 1 Institute of Biomedical Materials and Engineering, College of Materials Science and Engineering, College of Chemistry and Chemical Engineering, Qingdao University, Qingdao 266071, China; wangyifan@qdu.edu.cn (Y.W.); lianghe169@163.com (L.H.); yubingqdu@yahoo.com (B.Y.); cyalex1995@163.com (Y.C.); amnano@163.com (Y.S.); 2 Laboratory for New Fiber Materials and Modern Textile, Growing Base for State Key Laboratory, Qingdao University, Qingdao 266071, China; 3 Key Laboratory of Biomass Chemical Engineering of Ministry of Education, Center for Bionanoengineering, and Department of Chemical and Biological Engineering, Zhejiang University, Hangzhou 310027, China

**Keywords:** Zinc oxide quantum dots (ZnO QDs), poly(carboxybetaine methacry late) (PCBMA), poly(2-(dimethylamino) ethyl methacrylate) (PDMAEMA), drug delivery

## Abstract

In this paper, we reported a pH responsive nano drug delivery system (NDDS) based on ZnO quantum dots (QDs) for controlled release of drugs. Zwitterionic poly(carboxybetaine methacrylate) (PCBMA) and poly(2-(dimethylamino) ethyl methacrylate) (PDMAEMA) were introduced to modify ZnO QDs, which can help enhance water stability, increase blood circulation time, and promote endocytosis. After tuning of PCBMA/PDMAEMA ratios, the ZnO@P(CBMA*-co-*DMAEMA) nanoplatform shows a sensitive switch from strong protein adsorption resistance (with negatively charged surface) at physiological pH to strong adhesion to tumor cell membranes (with positively charged surface) at the slightly acidic extracellular pH of tumors. Anti-cancer drug, Doxorubicin (DOX), molecules were demonstrated to be successfully loaded to ZnO@P(CBMA*-co-*DMAEMA) with a relatively large drug loading content (24.6%). In addition, ZnO@P(CBMA*-co-*DMAEMA) loaded with DOX can achieve lysosomal acid degradation and release of DOX after endocytosis by tumor cells, resulting in synergistic treatment of cancer, which is attributed to a combination of the anticancer effect of Zn^2+^ and DOX.

## 1. Introduction

Cancer is a serious threat to people’s health all over the world. Nowadays, cancer therapy relies mainly on chemotherapy and radiation therapy, which has great side effects along with great sequela [1,2]. Nano drug delivery systems (NDDSs) are commonly researched as a strategy for tumor treatments, which can help avoid damaging healthy cells, and improve cancer treatment routes [3,4]. Based on the enhanced permeability and retention (EPR) effect, NDDSs can reach to lesions, which are modified by one or two major types of protein resistant materials [5], polyethylene glycol (PEG) polymers [6,7] or zwitterionic polymers [8,9,10]. Unfortunately, the PEGylation of NDDS has a negative effect on the internalization of NDDS due to the strong protein adsorption resistance structure of PEG, thus reducing the therapeutic effect [11,12,13].

ZnO quantum dots (QDs) have drawn much attention because of the advantages of low cost, ease of availability, biocompatibility, and high thermal stability [14,15]. The unprotected ZnO QDs can be employed as nanocarriers for drug delivery as they can decompose completely to zinc ions at pH = 5 in aqueous solution [16,17]. In addition, zinc ions are toxic to tumor cells, which can help achieve synergistic cancer treatment with the delivered drugs [18]. However, there are several drawbacks of ZnO QDs, including poor water stability and easy agglomeration, which hinders its application in the biological domain [19,20,21]. In recent years, the use of functional modifications of ZnO NPs in the field of drug delivery systems has drawn much attention. According to previous reports, stable ZnO NPs can be modified with a polymer of methyl methacrylate (MMA) [22], polystyrene (PS) [23], oleic acid [24], folic acid [25], hyaluronic acid [26], and poly(2-(dimethylamino) ethyl methacrylate) (PDMAEMA) [27], etc. The zwitterionic polymers have been developed to enhance retention and internalization due to the difference of a mildly acidic extracellular pH in tumor tissues (pH = 6.5–6.9) [28,29] as compared to healthy tissue under physiological conditions (pH = 7.2–7.4) [30,31]. Poly(carboxybetaine methacrylate) (PCBMA), a zwitterionic polymer, has a similar structure to glycine betaine, which is a kind of solute used to osmotically regulate living organisms. It is attractive for many biomedical applications based on its biomimetic and non-toxic nature [32,33]. Furthermore, PCBMA-decorated surfaces help prevent non-specific protein adsorption, which have a positive influence on the internalization of the NDDS [34,35,36,37].

**Scheme 1:**
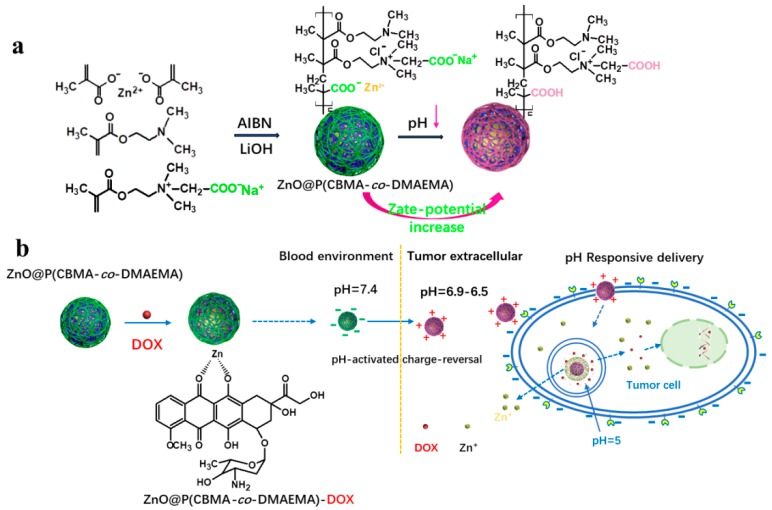
(**a**) Procedures to synthesize ZnO@P(CBMA*-co-*DMAEMA) NDDS; (**b**) schematic illustration of the ZnO@P(CBMA-*co*-DMAEMA) NDDS for drug delivery and deeply penetrating cancer therapy motivated by affinity switching.

In this study, we invented a pH responsive NDDS based on the ZnO QDs coated with zwitterionic polymer P(CBMA*-co-*DMAEMA) (Scheme 1a), which can help enhance water stability, increase blood circulation time, and promote endocytosis. Doxorubicin (DOX) was selected as a model drug for this study and was loaded to the NDDS by covalent interactions and formation of a Zn^2+^-DOX chelate complex. After ZnO@P(CBMA*-co-*DMAEMA) entering the cancer cells, lysosomal acid degradation can be achieved and the anticancer drug, DOX, is released, resulting in zinc ion synergistic treatment of cancer (Scheme 1b).

## 2. Materials and Methods

### 2.1. Materials

Zinc chloride, triethylene glycol (TEG), ethanol, and lithium hydroxide (LiOH·H_2_O) were purchased from Qingdao Renhe Xing Experimental Technology Co., Ltd. (Qingdao, China) and used without further purification. Methacrylic acid (MAA) was obtained from Aladdin (Shanghai, China) and distilled under reduced pressure prior to use. 2, 2-azobisisobutyronitrile (AIBN) was purchased from Qingdao Zhengye Experimental Technology Co., Ltd. (Qingdao, China) and recrystallized from ethanol. 2-(dimethylamino)ethyl methacrylate (DMAEMA, 97%). Doxorubicin (in the form of a hydrochloride salt) was obtained from Aladdin.

### 2.2. Synthesis of Zinc Dimethacrylate

Zinc dimethacrylate (Zn(MAA)_2_) was synthesized based on the reported method [38]. 3.2 g sodium hydroxide was dissolved in 20 mL deionized water, followed by 7.88 g methacrylic acid being added to the solvent. Then, it was sufficiently stirred to make sure reacting was completed. PH was tuned to 7.0 before adding ZnCl_2_ solution. Then, vacuum filtration was used to get the solid precipitate followed by washing for several times. Finally, the product was obtained after lyophilization.

### 2.3. Preparation of ZnO@P(CBMA-co-DMAEMA) Hybrid Nanoparticles with Different CBMA/DMAEMA Ratios

ZnO@P(CBMA-co-DMAEMA) was prepared by the sol-gel method [39]. First, 100 mg Zn(MAA)_2_ was dissolved in 20 mL TEG under magnetic stirring at 72 °C for 3 h. Then, the solution was cooled down to room temperature. Different amounts of CBMA, DMAEMA, and 5mg AIBN were added. The solution was stirred and heated at 72 °C for 10 min. Afterwards, 10 mg LiOH·H_2_O and another 5 mg AIBN were added into the reaction. Then, the reaction solution was kept stirring at 72 °C for 2 h. Finally, the solution was cooled down to room temperature and dialyzed against deionized water for 3 days.

### 2.4. Stability Tests of the ZnO-Based NDDSs

The resistance protein adsorption capability of ZnO NDDSs (1 mg mL^−1^) coated with different polymers (each condition has 5 samples) in 10% FBS solution were investigated with turbidimetry. The turbidity of samples was investigated at 600 nm by UV absorption spectrum at different time (0, 1, 2, 3, 4, 5, 6, 8, 10, 15 days).

### 2.5. Characterization

Fourier transform infrared (FT-IR) analysis was carried out using KBr discs in the region of 4000–500 cm^−1^ by a Fourier transform infrared spectrophotometer (Shimadzu IR prestige-21, Osaka, Japan). Ultraviolet–visible (UV-Vis) absorption spectra were performed on a Lambda 750 spectrophotometer (PerkinElmer, New York, NY, USA). The photoluminescence (PL) spectra were recorded on a Shimadzu RF-5301 PC spectrofluorophotometer (Osaka, Japan). The particle sizes of NPs were obtained by a Britain Malvern PSA (NANO2590, Beijing, China) submicron particle size analyzer with angle detection at 90°. The microstructure and morphology of the NPs were examined by high-resolution transmission electron microscopy (HRTEM, Germany, Berlin) on a Philips Tecnai G2F20 microscope (Philips, Amsterdam, Netherlands) with an accelerating voltage of 200 kV. The crystal structure of the NPs was investigated by XRD (Rigaku D/MAX-2400 X-ray diffractometer with Ni-filtered Cu Kα radiation (λ = 1.54056), Osaka, Japan).

### 2.6. Drug Loading and Release

0.5 mg mL^−1^ DOX solution (4 mL) was mixed with the solution of the ZnO-based (10 mg) NDDSs, followed by keeping it in the dark for 12 h. The drug loading content was calculated by UV-vis absorbance of DOX at 490 nm. The calibration curve obtained by a series of DOX solutions with different concentrations under the same conditions was used to be the reference system. In vitro release experiments were carried out at different pH values (PBS buffer). Firstly, 1 mL ZnO@PDMAEMA-DOX or ZnO@PC_400_D_400_-DOX was put in a dialysis bag (1000 Da). Then, the dialysis bag was immersed in 200 mL PBS buffer or acetate buffer, followed by stirring for 24 h. 1 mL sample was collected periodically. Meanwhile, each sample was replaced with the same volume of fresh buffer. UV absorption spectrum was used to measure the amount of released DOX of the samples.

### 2.7. Confocal Microscopy

Live cells were imaged by a laser scanning confocal microscope (Zeiss LSM 800, Germany, Berlin). Firstly, HepG2 cells were seeded onto the glass-bottomed culture dishes (35 mm, MetTek, Beijing, China) with a density of 5 × 10^4^ cells/dish, followed by incubating at 37 °C in 5% CO_2_ overnight. Then, the cells were mixed with ZnO@PC_400_D_400_ (20 μg mL^−1^) or ZnO@PC_400_D_400_-DOX (DOX 0.5 μg mL^−1^). After 4 h incubation, PBS was used to wash the cells 3 times to remove the ZnO@PC_400_D_400_ or ZnO@PC_400_D_400_-DOX complexes, which was adsorbed on the surface of the cell membrane. Finally, the laser scanning confocal microscope was used to image the cells under an excitation wavelength of 360 nm.

### 2.8. Cytotoxicity Analysis

The cytotoxicity of the NPs were assessed through MTT experiment. Firstly, the HepG2 cells were seeded in a 96-well plate at 2 × 10^4^ cells/well and incubated overnight. ZnO QDs and the corresponding increasing concentrations of Zn^2+^ ions (ZnCl_2_) from 2.5 to 40 μg mL^−1^ were incubated with HepG2 cells for 24 h. Similarly, free DOX and various concentrations (2.5 to 40 μg mL^−1^) of ZnO@PC_400_D_400_ and ZnO@PC_400_D_400_-DOX were also incubated with HepG2 cells for 24 h. Afterwards, the medium was replaced with 100 μL MTT (5 mg in 1 mL PBS and 9 mL FBS) and incubated for another 4 h. Finally, all medium was removed and 100 μL DMSO was added and shaken for 15 min. The absorbance of MTT at 490 nm was measured by an enzyme-labeled instrument. The cell viability (%) was calculated as:

(1) $$\;\mathrm{cell}\;\mathrm{viability}\;\left(\%\right)=(A_{S}/A_{C})\times 100\%\;$$

where *A_s_* is the absorbance of the sample well and *A_c_* is the absorbance of the control well. The data was calculated as the mean of 5 samples of each condition.

## 3. Results and Discussion

We prepared ZnO@P(CBMA-*co-*DMAEMA) NPs with Zn(MAA)_2_, DMAEMA and CBMA by the sol-gel method [40]. The process of synthesis is shown in Scheme 1a. The relevant parameters of NDDSs with different C(CBMA)/B(DMAEMA) ratios were depicted in Table 1. It is obvious that they show comparable hydrodynamic diameters (*D*_h_) with ca. 10 nm and a relatively small polymer dispersity index (PDI). 

**Figure 1:**
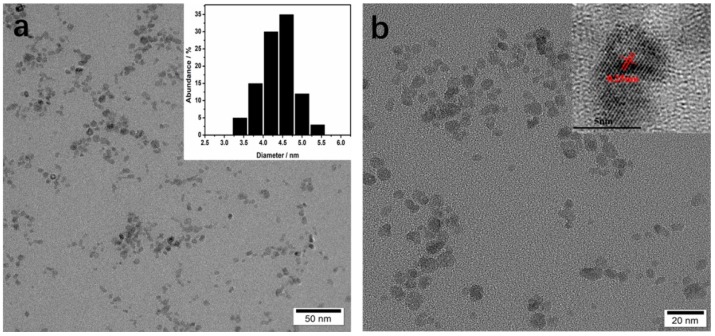
(**a**) TEM image and (inset) size distribution of ZnO@PC_400_D_400_NPs; (**b**) TEM image and (inset) HRTEM of ZnO@PC_400_D_400_NPs.

**Figure 2:**
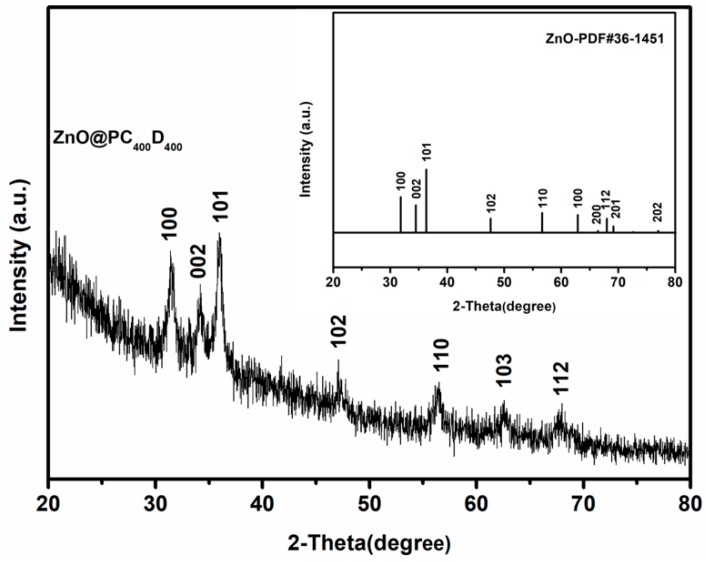
XRD patterns of ZnO@PC_400_D_400_NPs (the right upper inset was the standard pattern of ZnO (JCPDS 36-1451)).

The morphologies of the NDDSs were investigated by TEM. Figure 1 shows the TEM images of ZnO@PC_400_D_400_. The ZnO@PC_400_D_400_ NPs are uniform and monodispersed stably in water, exhibiting an approximate particle size of 6~10 nm. As in seen in Figure 1b, the averaged distance between the ZnO@ PC_400_D_400_ lattices is about 0.25 nm, indicating that adding PC_400_D_400_ does not affect the ZnO lattice. The crystal phase of ZnO nanoparticles was further identified by XRD. The XRD patterns of ZnO@PC_400_D_400_ NPs were well matched with the standard JCPDS data card of ZnO (Figure 2).

**Figure 3:**
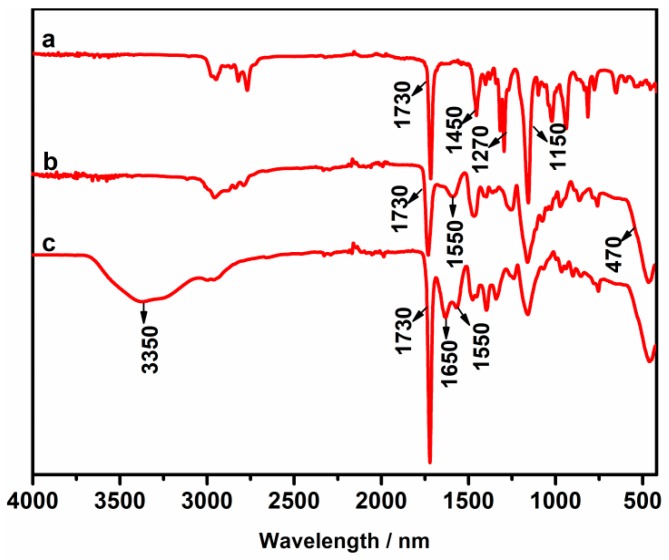
FTIR spectra of (a) PDMAEMA; (b) ZnO@PDMAEMA NPs, and (c) ZnO@PC_400_D_400_ NPs.

Figure 3 shows the FT-IR spectra of PDMAEMA, ZnO@PDMAEMA, and ZnO@PC_400_D_400_ samples. The absorption at 1730 cm^−1^ (–COOR), 1450 cm^−1^ (C–N), and 1150 cm^−1^ (C–O) are characteristic absorptions of PDMAEMA, which appeared in PDMAEMA homopolymer, ZnO@PDMAEMA, and ZnO@PC_400_D_400_ Compared to PDMAEMA homopolymer, ZnO@PDMAEMA NPs yield two new absorption bands located at 1550 cm^−1^ and 470 cm^−1^, which is attributed to unidentate coordination modes of acetate group with Zn [41] and the characteristic absorption peak of Zn–O. The results indicated that PDMAEMA was successfully coated on the surface of ZnO QDs. A new absorption peak at 1650 cm^−1^ was shown for ZnO@PC_400_D_400_, which belonged to the unidentate coordination modes of the acetate group with Na. Another new absorption peak appeared at 3350 cm^−1^, which indicates that COO– has been partially converted to COOH. The results indicate that ZnO@P(CBMA*-co-*DMAEMA) NPs was successfully prepared.

**Figure 4:**
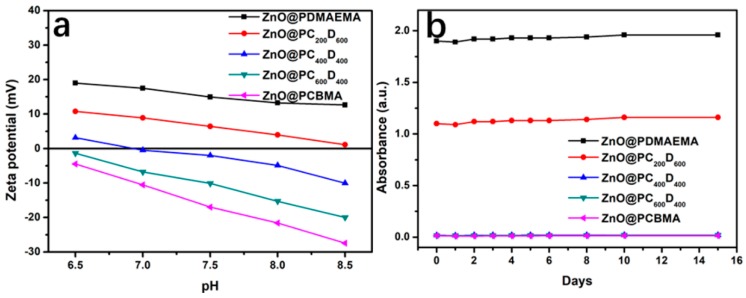
(**a**) Zeta potential of the ZnO NDDSs at different pH; (**b**) absorbance at λ = 600 nm showing the turbidity of the NDDSs incubated in 10% FBS with different times (0, 1, 2, 3, 4, 5, 6, 8, 10, and 15 days).

We tested the zeta potential of the NDDSs at different pH (Figure 4a, Table 2). As ZnO QDs can be dissolved under acidic conditions, the measuring pH was set from 6.5 (ZnO QDs will be dissolved a little, which is not enough to affect the final zeta-potential results) to 8.5. It is noticed for ZnO@PDMAEMA NDDS, the zeta potentials were both positive at pH = 6.5 and pH = 8.5, and decreased a little from pH = 6.5 to pH = 8.5. For ZnO@PC_200_D_600_ NDDS, the zeta potentials were also positive at both pH = 7.5 and pH = 6.5. However, as PCBMA content increased, the zeta potential dropped a lot compared to ZnO@PDMAEMA NDDS and decreased rapidly as the pH increased. This is attributed to that when in acidic environment, the number of negatively charged functional groups decrease due to the protonation of carboxyl groups on zinc carboxylate and CBMA, while the number of positively charged groups increase due to the protonation of the unprotonated tertiary amine group. This trend was the same as the CBMA moiety increases. For ZnO@PC_400_D_400_ NDDS, the zeta potential became negative (−0.21) at physiological pH (7.5), while positive (3.14) at the slightly acidic extracellular pH of tumors (6.5). The negative zeta potential at physiological pH could help resist protein adsorption and increase the circulation time. This was demonstrated by Figure 3b, in which the aggregation behavior of the NDDSs were investigated in 10% FBS to evaluate their protein adsorption behavior. The turbidity of ZnO@PDMAEMA dispersed solution reached a high value rapidly, indicating rapid aggregation of ZnO@PDMAEMA in 10% buffered FBS. In comparison, ZnO@PC_200_D_600_ with decreased positive charges showed less aggregation in 10% buffered FBS. When the zeta potential became negative (ZnO@PC_400_D_400_, ZnO@PC_600_D_200_, ZnO@PCBMA), almost no aggregation in 10% buffered FBS was observed for 15 days.

For ZnO@PC_400_D_400_ NDDS, the negative zeta potential at physiological pH and positive zeta potential at the slightly acidic pH not only helps resist protein adsorption, but also contributes to endocytosis. This is because the positively charged ZnO@PC_400_D_400_ NDDS in the acidic tumor microenvironment can be better attached to the negatively charged cell membrane, thus increasing endocytosis. In addition, the fast protonation/deprotonation of the ZnO@PC_400_D_400_ NDDS is reversible, which makes the NDDS much safer because the NDDS that do not enter the tumor by endocytosis will restore to their slightly negatively charged state to reduce endocytosis and internalization in healthy tissues. As the CBMA content kept increasing, the NDDSs became all negative at both pH = 7.5 and pH = 6.5, which was not favorable for endocytosis of tumor cells. As a result, ZnO@PC_400_D_400_ NDDS was selected for further research.

The thermogravimetric analysis (TGA) result of ZnO@PC_400_D_400_ NDDS is shown in Figure S1. It is seen that the polymers were almost completely decomposed when the temperature reached 400 °C. The weight fraction of ZnO in ZnO@PC_400_D_400_ was about 30 wt %. As is shown in Figure S2, the UV-Vis absorption spectra of the ZnO@PC_400_D_400_ NPs in aqueous solution stayed unchanged during the 30-days storage, proving that they are very stable in the neutral aqueous solution.

Doxorubicin (DOX) was selected as a model drug for this study. The drug loading content of ZnO@PDMAEMA, and ZnO@PC_400_D_400_ decided by UV-vis spectroscopy were 15.5% and 24.6% respectively. The relatively high loading content of drug molecules indicated the ZnO@PC_400_D_400_ nanoplatform is a promising candidate as NDDS. The lower drug loading content of ZnO@PDMAEMA resulted from like charges being repelled by the positively charged surface of DOX and ZnO@PDMAEMA. The loading content of ZnO@PC_400_D_400_ was much higher than that of ZnO@PDMAEMA, probably because of the zeta potential of ZnO@PC_400_D_400_ at physiological pH reducing to negative, which was more favorable for DOX loading.

**Figure 5:**
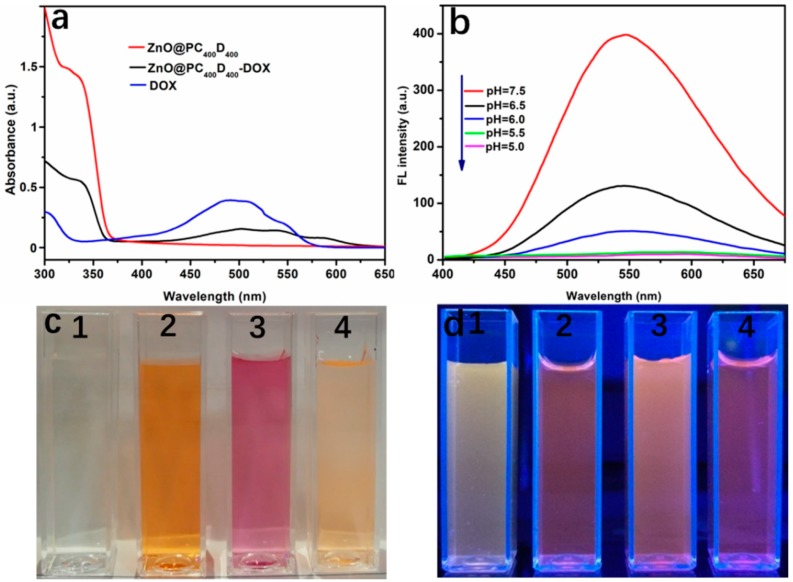
(**a**) UV−vis absorbance spectra of ZnO@PC_400_D_400_, ZnO@PC_400_D_400_-DOX, and free DOX; (**b**) fluorescence spectra of ZnO@PC_400_D_400_ incubated at different pH under an excitation of 330 nm. Photographs of aqueous solutions of (1) ZnO@PC_400_D_400_, (2) free DOX, (3) ZnO@PC_400_D_400_-DOX nanoparticles, and (4) DOX released from ZnO@PC_400_D_400_-DOX at pH = 5.0 under (**c**) white light and (**d**) UV light (365 nm).

Figure 5a is the UV−vis absorbance spectra of ZnO@PC_400_D_400_, ZnO@PC_400_D_400_-DOX and free DOX. The characteristic absorbance peak of ZnO@PC_400_D_400_-DOX at 500 nm (in accordance with free DOX) indicated that DOX was loaded to ZnO@PC_400_D_400_ successfully. There are two approaches for DOX to load into ZnO@PC_400_D_400_: (i) The complexation of DOX with Zn^2+^; (ii) the adsorption of positively charged DOX to negative ZnO@PC_400_D_400_ NDDS. The fluorescence spectra of ZnO@PC_400_D_400_ were detected after incubation with different pH solutions (Figure 5b). An emission peak at 550 nm was observed in physiological pH when excited at 330 nm, proving that ZnO QDs with yellow fluorescence were successfully synthesized. The emission peak of ZnO@PC_400_D_400_ vanished under mildly acidic conditions, confirming that ZnO QDs were efficiently decomposed in a weakly acidic environment. 

Figure 5c,d show the photographs of aqueous solutions of the NPs under white light and UV light. The aqueous solution of ZnO@PC_400_D_400_ is transparent and colorless under white light while it exhibits yellow fluorescence under the excitation (365 nm). The colour of the DOX solutions changes from orange to pink after mixing with ZnO@PC_400_D_400_. The solution colour recovered from pink to orange when the pH of the solutions of ZnO@PC_400_D_400_-DOX was tuned to below 5.5, suggesting breaking of the coordinate bond between the drug and the metal ion, with the DOX molecules released from the NDDS surfaces resultantly. Figure 5c,d show the photographs of aqueous solutions of the NPs under white light and UV light. The aqueous solution of ZnO@PC_400_D_400_ is transparent and colorless under white light while it exhibits yellow fluorescence under excitation (365 nm). The colour of the DOX solutions changes from orange to pink after mixing with ZnO@PC_400_D_400_. The solution colour recovered from pink to orange when the pH of the solutions of ZnO@PC_400_D_400_-DOX was tuned to below 5.5, suggesting breaking of the coordinate bond between the drug and the metal ion with the DOX molecules released from the NDDS surfaces resultantly.

**Figure 6:**
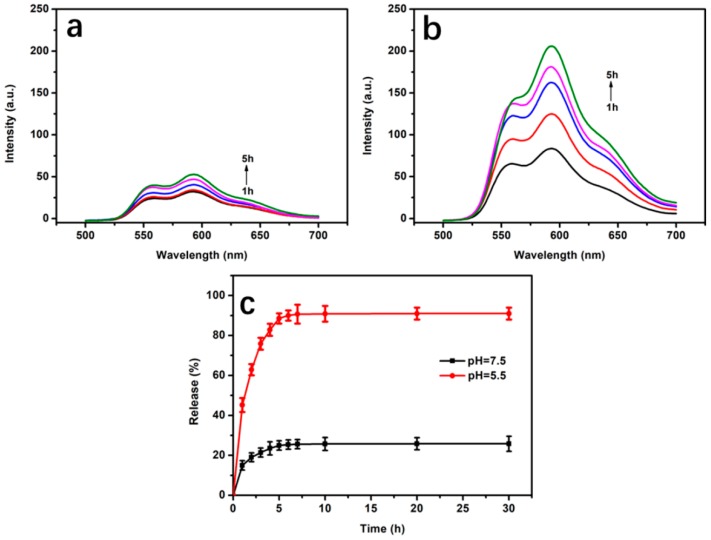
Time-dependent fluorescence spectra of drug release after incubating ZnO@PC_400_D_400_-DOX at (**a**) pH = 7.5 and (**b**) pH = 5.5 at different times (1−5 h); (**c**) the drug release profile of the ZnO@PC_400_D_400_-DOX on different pH environments.

pH-induced drug release behavior was investigated by fluorescence emission spectroscopy at different pH ranging from 1 to 5 h. As shown in Figure 6a, the fluorescence intensity of DOX increased a little after 5 h at pH = 7.5. In contrast, the fluorescence intensity of DOX increased rapidly in a short time (5 h) at pH = 5.5 (Figure 6b). The drug release study of ZnO@PC_400_D_400_-DOX was performed at different pH over 30 h (Figure 6c). It is observed that nearly 90% of DOX was released after 10 h in pH = 5.5 buffer, which simulated the physiological conditions of tumor cells. However, the amount of released DOX was no more than 25.3% in pH = 7.5 buffer, which simulated the physiological conditions of normal cells. The release of DOX was triggered by the decomposition of ZnO QDs and the drug-metal complex in an acidic environment.

**Figure 7:**
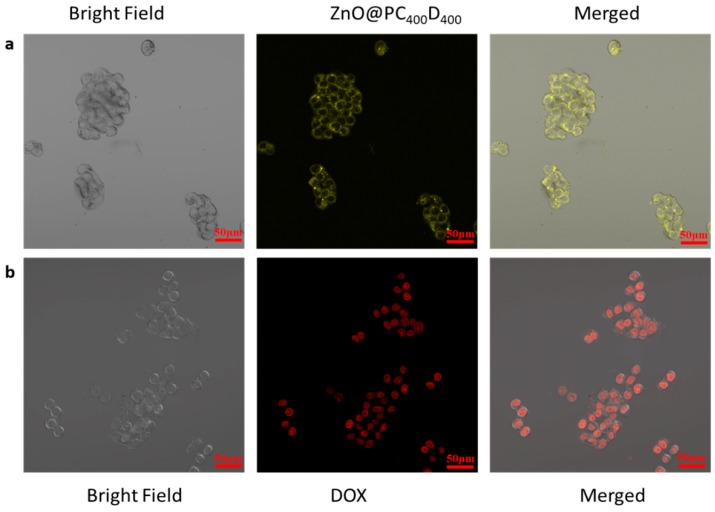
CLSM images in a culture of HepG2 cells of incubation with (**a**) ZnO@PC_400_D_400_ after 4 h and (**b**) ZnO@PC_400_D_400_-DOX after 8 h.

CLSM was conducted to investigate the internalization and drug release behaviors in tumor cells (Figure 7). Firstly, HepG2 cells were incubated with ZnO@PC_400_D_400_ and ZnO@PC_400_D_400_-DOX for 4 h and then subjected to CLSM observations. The endocytosis of ZnO@PC_400_D_400_ was monitored by the yellow fluorescence of ZnO QDs. As is seen in Figure 7a, the brightly yellow fluorescence in the cytoplasm clearly demonstrated the successful endocytosis of ZnO@PC_400_D_400_. The intracellular drug release from ZnO@PC_400_D_400_-DOX was tracked by the red fluorescence of DOX. In Figure 7b, the red fluorescence in HepG2 cells showed that DOX was successfully released from the NPs to the cells, which demonstrated that ZnO@PC_400_D_400_ NPs were decomposed in the intracellular acidic environment and promoted the drug release.

**Figure 8:**
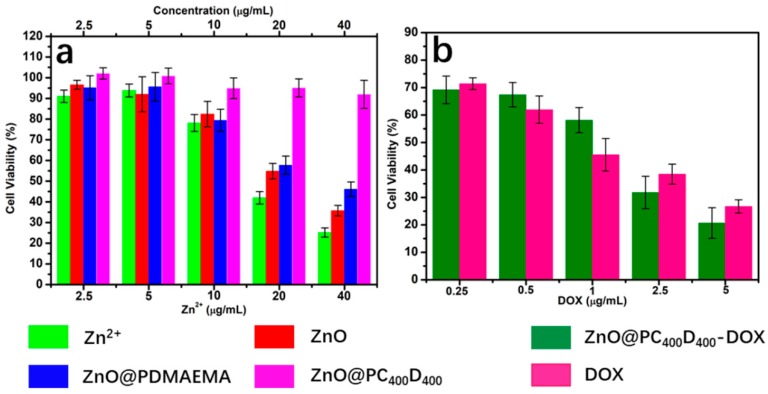
Cytotoxicity assay of HepG2 cells after 24 h of incubation with (**a**) ZnO QDs and comparable concentrations of Zn2+ ions, ZnO@PDMAEMA, and ZnO@PC_400_D_400_; (**b**) ZnO@PC_400_D_400_-DOX and free DOX.

The concentration-dependent cytotoxicity was evaluated with HepG2 cells. As is shown in Figure 8a, ZnO QDs and zinc ions (ZnCl_2_) both exhibited significant cytotoxicity, with zinc ions surpassing 20 μg mL^−1^, proving that ZnO QDs possessed comparable cytotoxicity effects after decomposition into free Zn^2+^. When the concentration of zinc ions become less than 10 μg mL^−1^, ZnO QDs can maintain more than 80% of cell viability. This is very important for zinc ions that are enriched in acidic cancer cells with cytotoxicity, while not to healthy cells. We also tested the cytotoxicity of the ZnO@PDMAEMA and ZnO@PC_400_D_400_. The results showed that at a concentration of 40 μg mL^−1^, more than 90% of HepG2 cells were viable for ZnO@PC_400_D_400_. This is due to that the negatively charged ZnO@PC_400_D_400_ encapsulating the ZnO QDs, which shields the inside QDs and reduces the cytotoxicity. However, when the concentration increased to 40 μg mL^−1^, only 45% of the cells in medium with addition of ZnO@PDMAEMA survived. This is because PDMAEMA is positively charged with cytotoxicity. As shown in Figure 8b, for the ZnO@PC_400_D_400_-DOX group, cell viability decreased to approximately 20.6% at 5 μg mL^−1^ DOX, which is even more cytotoxic than free DOX (with a cell viability of 26.7%), clearly proving DOX released in the acidic environment of tumors and the synergistic therapy of Zn^2+^.

## 4. Conclusions

In conclusion, we have created ZnO@P(CBMA*-co-*DMAEMA) NDDS with ZnO quantum dot as the fluorescence core and zwitterionic P(CBMA*-co-*DMAEMA) as the shell. The ZnO@P(CBMA*-co-*DMAEMA) NDDS possess excellent fluorescence properties for cell imaging and drug delivery. The surface protonation/deprotonation controlled charge switch of P(CBMA*-co-*DMAEMA) help increase blood circulation time and promote endocytosis. DOX were successfully loaded to ZnO@P(CBMA*-co-*DMAEMA) with a relatively large drug loading content (24.6%). The NDDS remained stable at physiological pH while it decomposed to release DOX under acidic intracellular conditions, resulting in synergistic treatment of cancer due to incorporation of the antitumor effect of Zn^2+^ and DOX. These advantages make ZnO@P(CBMA*-co-*DMAEMA) NDDSs a desirable unique drug delivery system for cancer treatment.

## Figures and Tables

**Table 1:** Structural parameters of different NDDSs.

Sample	CBMA/DMAEMA (mg/mg)	*D*_h_^1^ (nm)	PDI ^1^
ZnO@PDMAEMA	0/800	10.1	0.272
ZnO@PC_200_D_600_	200/600	10.6	0.318
ZnO@PC_400_D_400_	400/400	11.7	0.386
ZnO@PC_600_D_200_	600/200	11.6	0.324
ZnO@PCBMA	800/0	11.9	0.354

^1^*D*_h_ and PDI were measured by DLS.

**Table 2:** Zetal potential and drug loading contents of the NDDSs.

Sample	Zeta-Potential (mV) (pH = 7.5)	Zeta-Potential (mV) (pH = 6.5)
ZnO@PDMAEMA	14.94	18.99
ZnO@PC_200_D_600_	6.42	10.78
ZnO@PC_400_D_400_	−2.01	3.14
ZnO@PC_600_D_200_	−10.12	−1.38
ZnO@PCBMA	−16.98	−4.48

## Supplementary Materials

The following are available online at http://www.mdpi.com/2073-4360/10/11/1272/s1, Figure S1: Thermogravimetric Analysis (TGA) of ZnO@PC_400_D_400_, Figure S2: UV-Vis absorption spectra of ZnO@PC_400_D_400_ at different storage time at room temperature.
