# Supplementary material for: ZnO Quantum Dots Modified by pH-Activated Charge-Reversal Polymer for Tumor Targeted Drug Delivery
Source: Polymers (Basel). 2018 Nov 15;10(11):1272. doi: 10.3390/polym10111272 (PMC6401959; doi:10.3390/polym10111272)
Supplement: Supplementary file 1 [file polymers-10-01272-s001.doc]

**Supplementary Materials: ZnO Quantum Dots Modified by pH-Activated Charge-Reversal Polymer for Tumor Targeted Drug Delivery**

Yifan Wang, Liang He, Bing Yu, Yang Chen, Youqing Shen and Hailin Cong


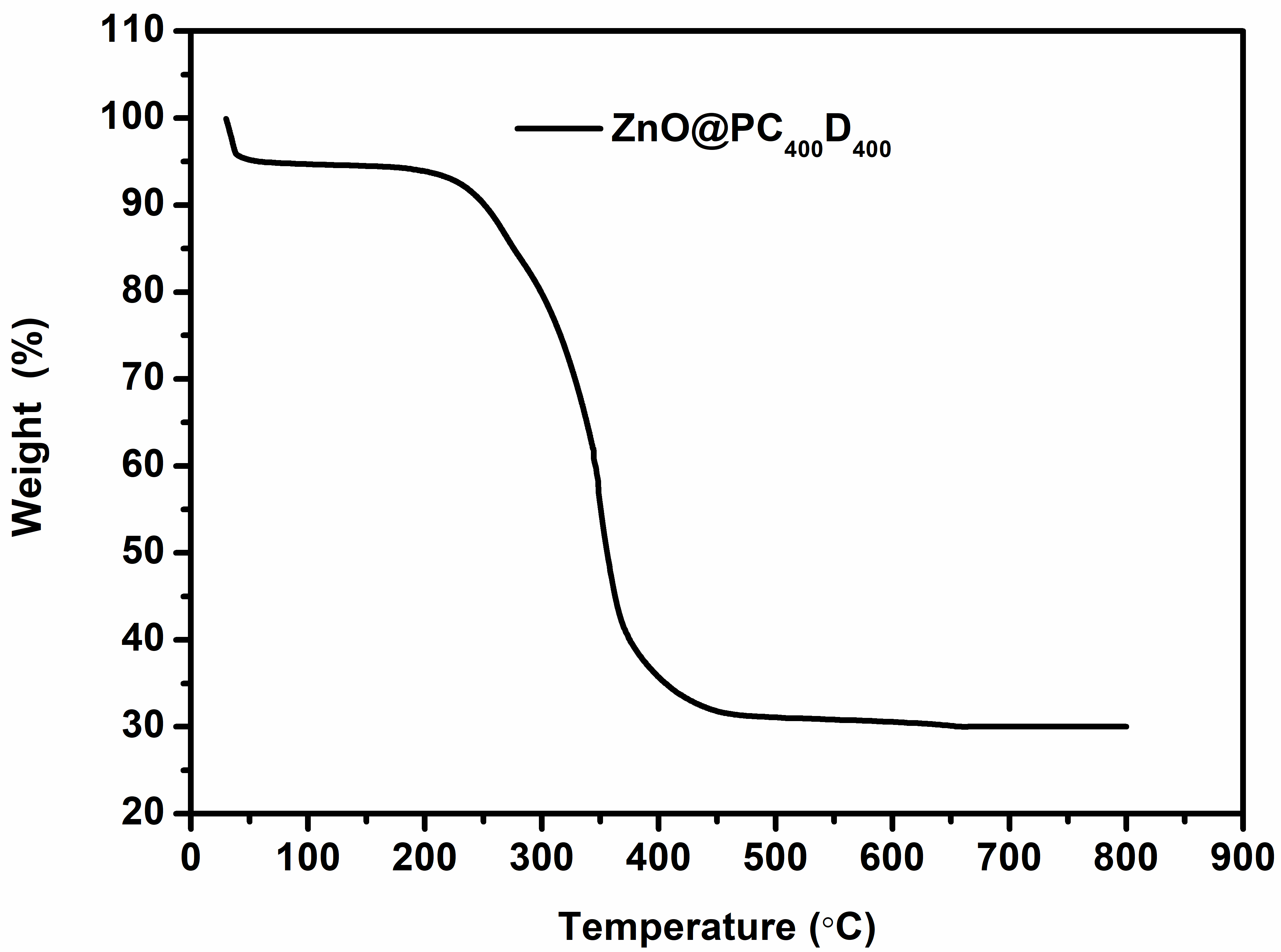


**Figuer S1**. Thermogravimetric Analysis (TGA) of ZnO@PC_400_D_400。_


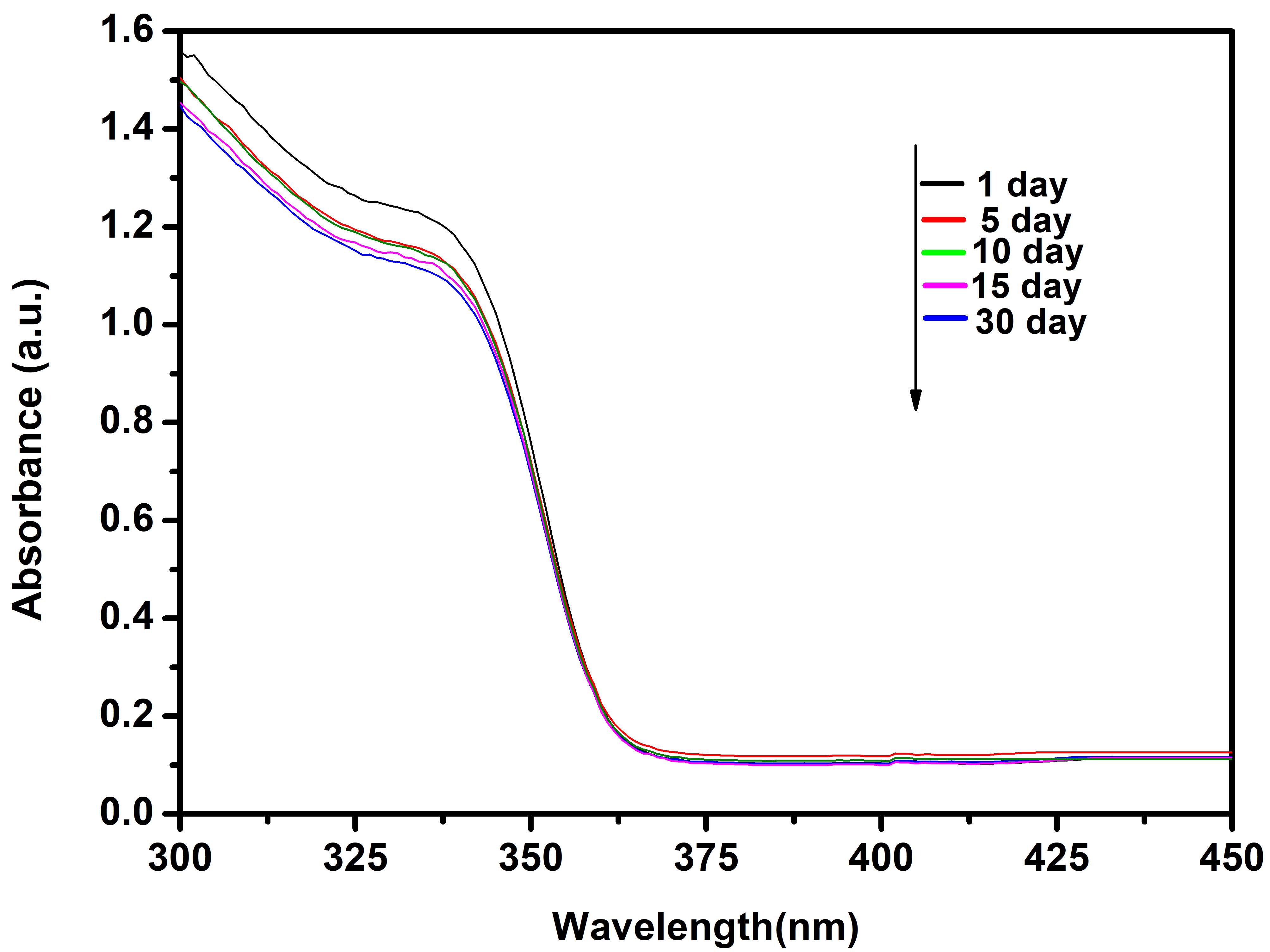


**Figure S2.** UV-Vis absorption spectra of ZnO@PC_400_D_400_ at different storage time at room temperature.
